# Risk-informed credibility goals for finite element simulation of rib bone plates: a case study in applying ASME V&V 40 to ASTM F382 testing

**DOI:** 10.3389/fbioe.2026.1875854

**Published:** 2026-10-26

**Authors:** Jun Sung Park, Chiseung Lee

**Affiliations:** 1 Department of Biomedical Engineering, Graduate School, Pusan National University, Busan, Republic of Korea; 2 Department of Biomedical Engineering, School of Medicine, Pusan National University, Busan, Republic of Korea; 3 In Silico Medicine Lab, Biomedical Research Institute, Pusan National University Hospital, Busan, Republic of Korea

**Keywords:** ASME V&V 40, credibility assessment, finite element analysis, four-point bending, rib bone plate, uncertainty quantification

## Abstract

**Introduction:**

While Computational Modeling and Simulation (CM&S) holds significant potential as key evidence in the design and regulatory approval process for medical devices, establishing the credibility of these results remains paramount. This study presents a case study applying the ASME V&V 40 risk-based framework to the evaluation of the four-point bending performance of rib bone plates.

**Methods:**

We established two distinct Contexts of Use (COUs) for a Class III rib bone plate. COU 1 (Design Change) utilizes CM&S as stand-alone evidence for a design change (increasing the number of holes), corresponding to a High-Medium model risk. Conversely, COU 2 (Product Line Extension) employs CM&S as supplementary evidence for product line expansion (varying bending shapes), corresponding to a Medium-Medium model risk. To meet the credibility requirements commensurate with each COU, we systematically performed Numerical Code Verification (NCV), calculation verification (discretization and solver errors), sensitivity analysis, Uncertainty Quantification (UQ), and validation of the Finite Element Analysis (FEA) model using ASTM F382 bench testing.

**Results:**

Verification activities confirmed numerical stability with an NCV error of 0.704%. Validation results demonstrated that the FE model agreed with experimental four-point bending results within 5% error. For key Quantities of Interest such as bending stiffness, the validation metric (|E|/Uval < 1) further supported model adequacy under the stated Context of Use.

**Discussion:**

Consequently, in COU 1, where the Question of Interest (QOI) is whether increasing the hole count degrades performance, the model predicted a negligible impact (<0.2%), illustrating how CM&S may support regulatory decisions on such design changes; as this prediction was not accompanied by physical testing of the extended configurations, experimental confirmation would provide complementary evidence. In COU 2, where the QOI is whether the model can predict the performance of new pre-contoured shapes, errors within the 10% threshold established for this COU support the use of the model as supplementary evidence. This case study illustrates an approach to defining the role of CM&S based on risk and tailoring corresponding VVUQ activities to secure the credibility of data submitted for medical device regulations.

## 1 Introduction

Computational Modeling and Simulation (CM&S), particularly Finite Element Analysis (FEA), has established itself as an indispensable tool in the design, development, and regulatory approval processes for medical devices (Morrison et al., 2017; 2018). In the field of orthopedic implants, FEA is extensively employed to evaluate mechanical performance and predict potential failure modes (Rana et al., 2020; Baumann et al., 2021; Parr et al., 2021). Because rib fractures are critical indicators of trauma severity (Flagel et al., 2005; Peek et al., 2020), their surgical treatment has received increasing attention. In particular, pre-contoured plates are now favored over intraoperatively contoured ones, because manual bending during surgery not only prolongs the procedure but may also introduce micro-cracks or residual stresses that compromise the mechanical integrity of the implant (Bottlang et al., 2010). Given the critical nature of these devices, classified as Class III by regulatory authorities such as the US Food and Drug Administration (FDA) and the Korean Ministry of Food and Drug Safety (MFDS) (Morrison et al., 2018; MFDS, 2025), rigorous performance data is mandatory for market approval (FDA, 2016). This evaluation is typically conducted via the four-point bending test specified in ASTM F382, which serves as the industry standard for characterizing the mechanical properties of metallic bone plates (ASTM International, 2017).

This aligns with the current global paradigm shift in regulatory science, which aims to reduce the burden of animal and human trials by strategically elevating high-quality *in silico* data to the level of pivotal evidence. Nevertheless, for CM&S results to be accepted as a valid replacement or complement to physical testing, establishing their credibility is paramount (FDA, 2023). To address this, the CM&S community has developed guidelines for Verification and Validation (V&V) (ASME, 2009; 2016). Notably, the ASME V&V 40 standard provides a risk-based framework that scales the rigor of V&V activities according to model risk, which is defined as the combination of two paired concepts. The first is Model Influence, which refers to the degree to which the computational model contributes to the regulatory decision relative to other evidence. The second is Decision Consequence, which refers to the severity of adverse outcomes that may result from an incorrect decision (ASME, 2018). Recent benchmark studies have begun translating this framework into practice for orthopedic devices, including FDA-regulated multicenter studies on spinal rods (Nagaraja et al., 2020) and an end-to-end ASME V&V 40 application to a pedicle screw system (Nagaraja et al., 2024). However, these foundational studies have primarily examined a single Context of Use, leaving an underexplored question regarding how the rigor of credibility activities should be scaled and tailored when the same computational model is intended to serve multiple regulatory roles with differing Model Influence but comparable Decision Consequence. This question is particularly relevant within the regulatory landscape of the Korean Ministry of Food and Drug Safety (MFDS), where CM&S has traditionally played a supportive role (Lee et al., 2024) and where practical guidance on risk-informed credibility scaling remains limited (Scully et al., 2018; Morrison et al., 2019; Berti et al., 2021; Santiago et al., 2022; Aldieri et al., 2023; Curreli et al., 2023; Jeong et al., 2025).

To address this gap, this study applies the ASME V&V 40 framework to rib bone plates, a Class III trauma device not previously addressed in published V&V case studies. Building upon the end-to-end methodology established by prior orthopedic V&V work (Nagaraja et al., 2020; 2024), the central contribution of this study is to demonstrate how credibility activities can be risk-informed and tailored across two distinct Contexts of Use (COUs) within a single coherent study. Because both COUs concern the same Class III device, the Decision Consequence is held constant at Medium, while the Model Influence is varied to isolate the effect of the model’s regulatory role on credibility requirements. Specifically, COU 1 utilizes CM&S as stand-alone evidence for a design change (High Model Influence, yielding an overall model risk of High Model Influence and Medium Decision Consequence), whereas COU 2 utilizes the same validated model as supplementary evidence for product line expansion (Medium Model Influence, yielding an overall model risk of Medium Model Influence and Medium Decision Consequence). By contrasting the VVUQ requirements and outcomes of these two COUs side by side, we provide practical insights into how the paired axes of Model Influence and Decision Consequence translate into differentiated credibility activities for a single validated model. To our knowledge, this aspect has not been explicitly examined in prior end-to-end ASME V&V 40 case studies for orthopedic implants. It should be noted at the outset that the credibility established in this study applies specifically to titanium rib bone plates evaluated under the ASTM F382 four-point bending configuration, and to geometries within the plate family validated in this study. The applicability domain of the resulting model is therefore intentionally narrow, and any extension beyond this validated domain would require additional credibility activities, as discussed later in this manuscript.

## 2 Materials and methods

This study adopts the credibility assessment framework presented in the ASME V&V 40-2018 standard to establish the credibility of the computational model for the medical device. For risk analysis and the evaluation of credibility factors, we applied the gradation system recommended by the standard.

### 2.1 Question of interest and context of use

To establish the credibility requirements for the medical device computational model, we first defined the Question of Interest (QOI) and Context of Use (COU) for the two cases mentioned in the Introduction, in accordance with the ASME V&V 40-2018 standard.

#### 2.1.1 Question of interest

The Question of Interest (QOI) defines the specific question the model is intended to answer. For COU 1, the QOI is whether a design modification, such as changing the number of holes, degrades key mechanical performance compared to the existing design under the ASTM F382 static four-point bending test criteria. For COU 2, the QOI is whether the validated model can accurately predict the mechanical performance of new geometries, such as pre-contoured 8-hole plates. The Quantities of Interest (QOIs) are the model output variables used to address the QOI, corresponding to the mechanical performance metrics defined by ASTM F382.

#### 2.1.2 Context of use

COU 1 corresponds to the case where CM&S is utilized as stand-alone evidence for a design change. The Context of Use is defined as using the model framework, validated against bench test results for the existing design, to simulate the performance of the modified design and answer the QOI without additional physical testing. Under this COU, the simulation results function as the only source of evidence answering the QOI.

COU 2 corresponds to the case where CM&S is utilized as supplementary evidence for product line expansion and verification. The COU involves performing bench tests on the pre-contoured 8-hole plates alongside model predictions to cross-compare and verify the CM&S results against physical test data. In this case, while the model’s prediction provides important information, the final regulatory decision relies primarily on experimental data.

### 2.2 Model risk assessment

We followed the risk-based framework defined in ASME V&V 40-2018 to establish the credibility requirements for the medical device computational model. Model Risk is defined as the combination of Model Influence and Decision Consequence. This combination determines the required level of credibility for Verification, Validation, and Uncertainty Quantification (VVUQ) activities (ASME, 2018; 2022). Model Influence refers to the extent to which the computational model contributes to the decision-making process relative to other contributing evidence, such as bench test results. Decision Consequence refers to the significance of the adverse outcomes that could result from an incorrect decision regarding the question of interest.

#### 2.2.1 Decision Consequence

The rib bone plate is classified as a Class III medical device under the Korean MFDS four-tier classification system, corresponding to an upper-middle risk category, with Class IV representing the highest risk. Incorrect decisions, such as the mechanical failure of the rib bone plate, can lead to adverse patient outcomes. Since deformation or failure of the plate has been shown to cause patient injury or the need for reoperation, this corresponds to the ASME V&V 40 guideline description where an incorrect decision leads to minor patient injury, the need for medical intervention, or other moderate impacts. Therefore, in this study, the Decision Consequence associated with the mechanical performance evaluation of the rib bone plate was selected as Medium.

#### 2.2.2 Model Influence

Based on the COUs defined in Section 2.1.2, the Model Influence levels were evaluated for each case. COU 1 represents a case of High Model Influence. Since the CM&S output acts as a determinant factor in the decision, the Model Influence level was set to High. COU 2 represents a case of Medium Model Influence. Therefore, the model performs a supplementary role, and the Model Influence level was set to Medium.

#### 2.2.3 Overall model risk and credibility goals

By combining the defined Model Influence and Decision Consequence, the Overall Model Risk for each COU was derived (Figure 1). The detailed definitions and classification system for model risk grades are presented in Supplementary Appendix SA (Supplementary Table SA1). Although the Decision Consequence for the rib bone plate (Class III medical device) was Medium for both COUs, the overall risk and required credibility levels were differentiated based on the role of the model, or Model Influence. The credibility activities for COU 2 were therefore performed at the more rigorous level required for COU 1, while the acceptance criterion for each output comparison was assigned according to the respective model risk.

**FIGURE 1:**
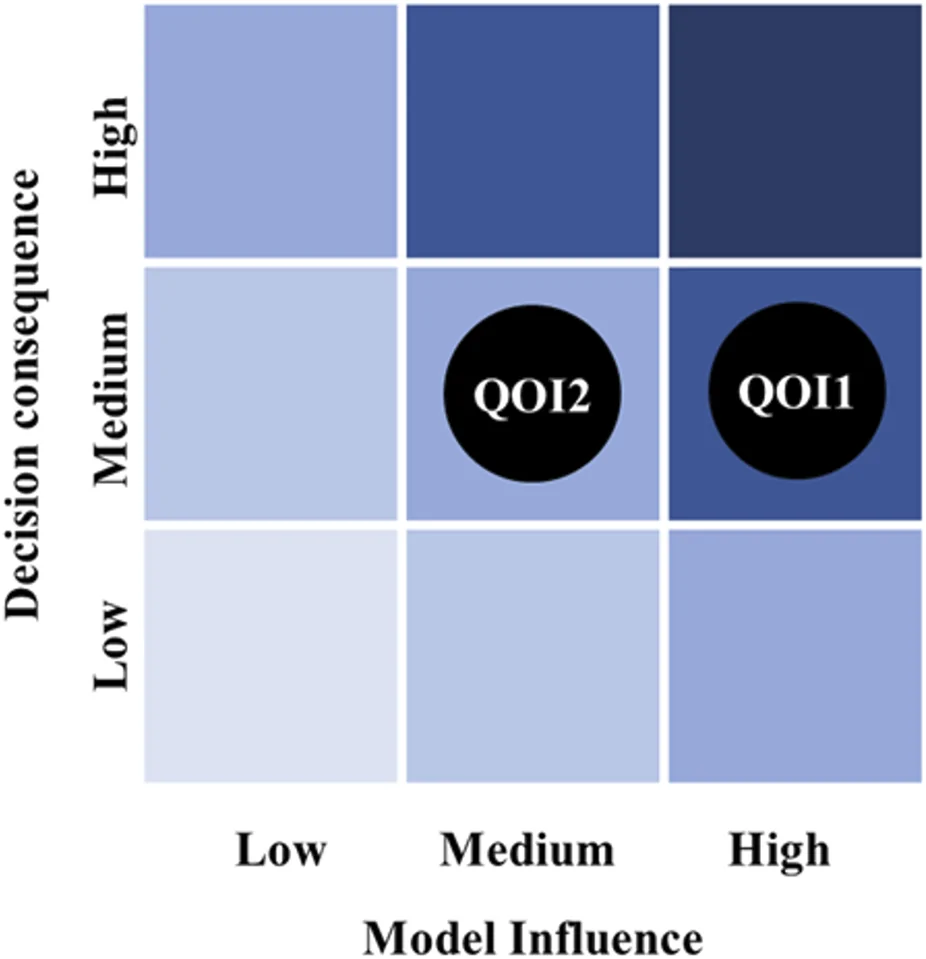
Model risk matrix based on ASME V&V 40, showing the positions of the two Contexts of Use considered in this study. COU 1 (Model Influence: High, Decision Consequence: Medium) falls in the M-H region, whereas COU 2 (Medium, Medium) falls in the M-M region. A unified credibility framework calibrated to the higher-risk COU (M-H) was applied to both COUs, since the credibility grades in ASME V&V 40 are hierarchical and higher-tier activities inherently satisfy lower-tier requirements.

In COU 1, as the Model Influence was set to High, the Overall Model Risk was determined to be High-Medium. This implies that strict credibility requirements must be met for VVUQ activities. The corresponding gradation goals are established in Section 2.3. Conversely, in COU 2, since the Model Influence is Medium, the Overall Model Risk is set to Medium-Medium, requiring a relatively lower level of credibility activities compared to COU 1. In principle, a credibility framework could be constructed for each COU according to its respective risk level. In this study, however, a single credibility framework calibrated to the higher-risk COU (COU 1, High-Medium) was applied to both COUs. This choice was made because the credibility grades defined in ASME V&V 40 are hierarchical—activities that satisfy a higher grade inherently satisfy lower grades—and therefore a framework designed for High-Medium simultaneously accommodates the requirements of Medium-Medium. Accordingly, the credibility factor analysis in Section 2.3 was performed with respect to the High-Medium model risk.

### 2.3 Credibility factor analysis

Credibility factors are defined in the ASME V&V 40-2018 standard as individual activities related to establishing model credibility. In this study, considering the risk assessment results, a total of 23 credibility factors were evaluated. According to the standard, the number of gradations associated with each credibility factor ranges from 2 to 4. That is, some factors have 2 gradations (a to b), some have 3 (a to c), and others have 4 (a to d). The credibility level was subdivided into 12 equal vertical increments to map precisely to the number of gradations assigned to each factor in Table 1. Professional judgment was used to select and justify the gradation levels, with the goal of selecting gradations that meet the requirements of the High-Medium model risk. Accordingly, we established goals of level (b) or (c) for credibility factors with 3 gradations, and level (c) or (d) for those with 4 gradations. As shown in Table 1, the credibility grades adopted for COU 1 and COU 2 are identical, because the M-H framework designed for COU 1 was applied to both COUs. The definition of each selected grade is therefore summarized as a single grade per factor in Table 2, to aid interpretation without cross-referencing the appendix.

**TABLE 1:** Summary of credibility factors and target goals established for this study.

Credibility factor			Credibility level					
Verification	Code verification	Software quality assurance	c/c		​		​	
		Numerical code verification	c/d	​		​		​
	Calculation verification	Discretization error	b/c		​		​	
		Numerical solver error	c/c		​		​	
		Use error	c/d	​		​		​
Validation	Computational model	Model form	b/c		​		​	
		Model inputs: Quantification of sensitivities	b/c		​		​	
		Model inputs: Quantification of uncertainties	b-c/c	​		​		​
	Comparator	Quantity of test samples	b/c		​		​	
		Range of characteristics of test samples	b/d	​		​		​
		Measurements of test samples	b/c		​		​	
		Uncertainty of test sample measurements	c/d	​		​		​
		Quantity of test conditions	a/c		​		​	
		Range of test conditions	a/d	​		​		​
		Measurements of test conditions	b/c		​		​	
		Uncertainty of measurements of test conditions	c/d	​		​		​
	Assessment	Equivalency of input parameters	c/c		​		​	
		Output comparison: Quantity	b/b			​		
		Output comparison: Equivalency of output parameters	c/c		​		​	
		Output comparison: Rigor of output comparison	d/d	​		​		​
		Output comparison: Agreement of output comparison	b/c		​		​	
Applicability		Relevance of the QOIs	c/c		​		​	
		Relevance of the validation activities to the COU	c/d	​		​		​
​			L	L-M	M		M-H	H

**TABLE 2:** Definitions of the selected credibility grades listed in Table 1, excerpted from Supplementary Appendix SA (Supplementary Tables SA2–SA4). Since the credibility grades were identical for COU 1 and COU 2 in this study, a single grade is presented for each factor.

Credibility factor	Grade	Definition of the selected grade
Software quality assurance	c	SQA procedures were specified and documented; the software anomaly list and development environment are fully understood; the impact on the COU is analyzed and documented; and quality metrics are tracked
Numerical code verification	c	Discretization error was quantified through comparison with an exact solution, and a grid convergence study demonstrated that the numerical solution asymptotically approached the exact solution as the discretization was refined
Discretization error	b	Applicable grid convergence analyses were performed and their respective convergence behaviors were observed to be stable, but the discretization error was not estimated
Numerical solver error	c	Problem-specific sensitivity study was performed on solver parameters, confirming that changes in simulation results due to changes in the solver parameters were negligible relative to the model accuracy goal
Use error	c	Key inputs and outputs were verified by internal peer review
Model form	b	Influence of expected key model form assumptions was explored
Model inputs: quantification of sensitivities	b	Sensitivity analysis on expected key parameters was performed
Model inputs: quantification of uncertainties	b–c	Uncertainties on expected key inputs were identified and quantified, and were propagated to quantitatively assess the effect on the simulation results
Quantity of test samples	b	Multiple samples were used, but not enough to be statistically relevant
Range of characteristics of test samples	b	Samples representing a range of characteristics near nominal were included
Measurements of test samples	b	One or more key characteristics of the test samples were measured
Uncertainty of test sample measurements	c	Uncertainty analysis incorporated instrument accuracy and repeatability (i.e., statistical treatment of repeated measurements)
Quantity of test conditions	a	A single test condition was examined
Range of test conditions	a	A single test condition was examined
Measurements of test conditions	b	One or more key characteristics of the test conditions were measured
Uncertainty of test condition measurements	c	Uncertainty analysis incorporated instrument accuracy and repeatability (i.e., statistical treatment of repeated measurements)
Equivalency of input parameters	c	The types and ranges of all inputs were equivalent
Output comparison: quantity	b	Multiple outputs were compared
Output comparison: equivalency of output parameters	c	Types of outputs were equivalent
Output comparison: rigor of output comparison	d	Uncertainties in the output of both the computational model and the comparator were used in the output comparison
Output comparison: agreement of output comparison	b	The level of agreement of the output comparison was satisfactory for key comparisons, but not all comparisons
Relevance of the QOIs	c	The QOIs from the validation activities were identical to those for the COU.
Relevance of the validation activities to the COU	c	The COU encompassed some of the validation points

Full definitions of all grades (a–d) are provided in Supplementary Appendix SA (Supplementary Tables SA2–SA4).

### 2.4 Verification

#### 2.4.1 Code verification

Code verification is the stage of confirming that the numerical implementation of the software is correct and that the developed code operates accurately as intended. This verification is performed through Software Quality Assurance (SQA) of the simulation software and Numerical Code Verification (NCV). Detailed verification methods and results are provided in Supplementary Appendix SB.

#### 2.4.2 Calculation verification

Calculation verification is the process of confirming that the simulation software has accurately implemented the developed mathematical model, analyzing numerical errors, and ensuring the credibility of the results. Calculation verification is performed through the analysis and evaluation of Discretization Error, while the analysis and evaluation of Numerical Solver Error are covered in Supplementary Appendix SB.

##### 2.4.2.1 Discretization error verification

Discretization error verification was conducted to determine the effect of the model’s mesh size on the simulation results of the rib bone plate four-point static bending test. We verified the discretization error of the 8-hole rib bone plate model through mesh convergence studies under four-point static bending boundary conditions corresponding to ASTM F382 and a 50 N load condition. All components of the rib bone plate were modeled with quadratic tetrahedral elements ensuring consistent meshing. Analyses were performed for a total of 5 element sizes ranging from 0.25 mm to 1 mm.

### 2.5 Validation and applicability

Detailed credibility assessment according to ASME V&V 40-2018 is described in Supplementary Appendix SA. Within the appendix, Supplementary Table SA2 outlines the goals for credibility factors associated with Verification, while Supplementary Table SA3 presents the goals for credibility factors associated with Validation. Furthermore, Supplementary Table SA4 summarizes the goals for credibility factors associated with Applicability to the COU.

#### 2.5.1 FE model

The FE model of the Flat 8H plate, which features four holes on each side, was implemented via reverse engineering using an HDI Advance 3D Scanner and FlexScan3D. Considering the comprehensive trade-off between the data precision limitations inherent in reverse engineering and the efficiency of FEA, the internal thread geometry of the screw holes was excluded from the modeling and simplified to a cylindrical geometry. As shown in Figures 2A–C, the four-point static bending test corresponding to ASTM F382 was simulated by fixing the support rollers and applying axial displacement to the loading rollers. The support/loading rollers were determined considering the specifications of ASTM F382. Material properties for each component and initial simulation conditions were set as shown in Table 3 (Riekehr et al., 2020). The contact between the bone plate and the rollers was defined as surface-to-surface frictional contact using the Augmented Lagrangian formulation with the Nodal-Normal to Target detection method. The normal stiffness factor (FKN) was set to 1.0, and the remaining contact settings were adopted as program defaults. The friction coefficient was set to a baseline value of 0.1 based on the sensitivity analysis reported in Section 3.2.1.

**FIGURE 2:**
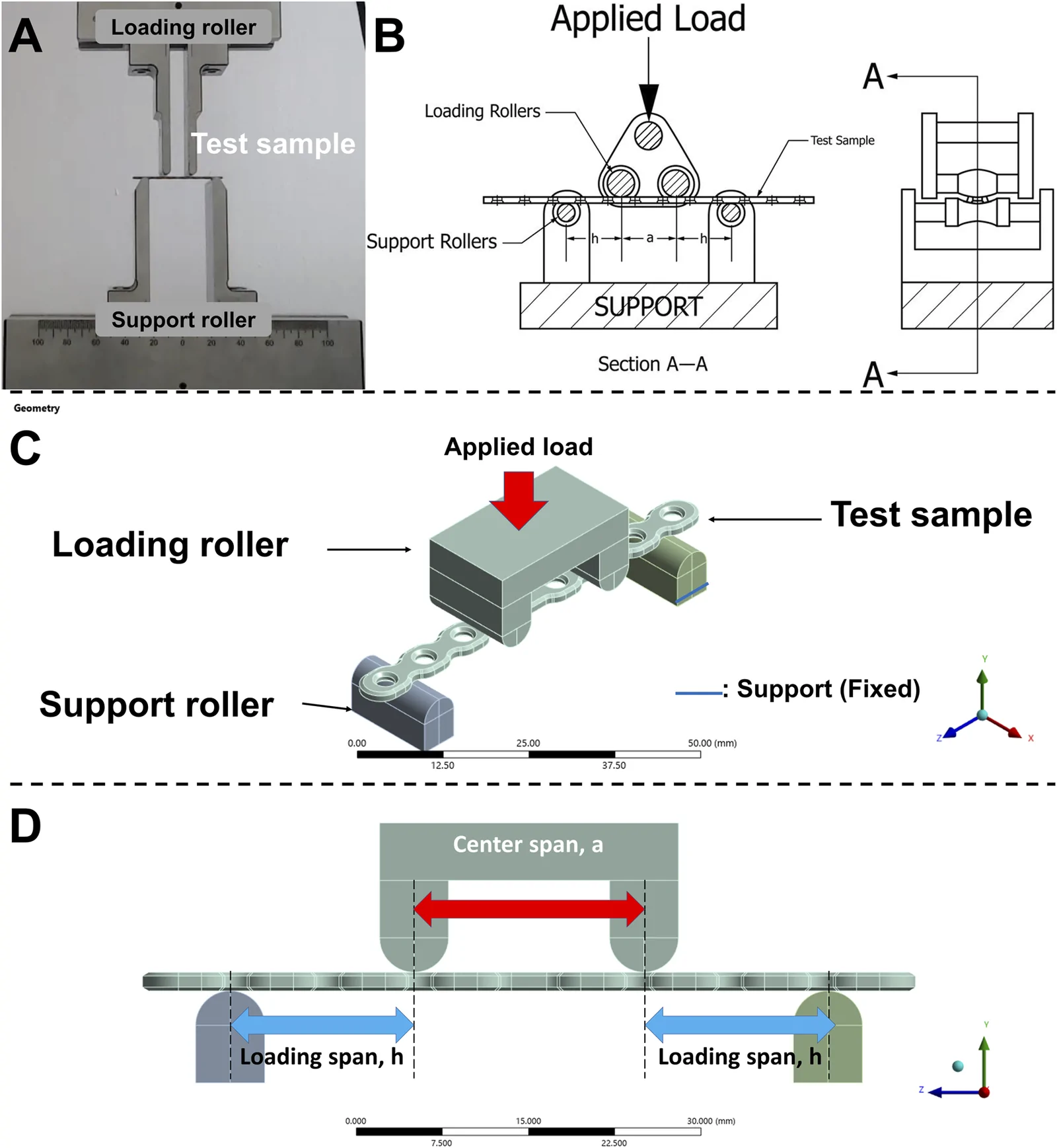
Schematic and experimental setup of the four-point bending test. **(A)** Experimental jig showing the loading and support rollers. **(B)** Schematic diagram illustrating the standard test geometry as defined in ASTM F382 (ASTM International, 2017). **(C)** Finite Element model configuration showing the applied load and fixed support. **(D)** Definition of input parameters for the FE model, indicating the loading span ( h ) and center span ( a ).

**TABLE 3:** Summary of material properties and simulation setup parameters.

Category	Parameter		Value/setting
General	Software and version		Ansys Mechanical 2024 R2
	Analysis type		Implicit
	Large deformation		On
Model	Simplifications		Screw threads simplified to cylindrical geometry
	Model geometry		Full model
Material properties	Bone plate	Name	Titanium grade 4
		Behavior	Bilinear isotropic hardening
		Stiffness behavior	Flexible
		Young’s modulus (MPa)	98,500
		Poisson’s ratio	0.29
		Yield stress (MPa)	581
		Additional details	Tangent modulus = 983.8 MPa
	Rollers	Name	Structural steel
		Behavior	Linear elastic
		Stiffness behavior	Rigid
		Young’s modulus (MPa)	200,000
		Poisson’s ratio	0.3
Mesh	Bone plate elements		Tetrahedral, quadratic, 0.3 mm
	Roller elements		Quadrilateral dominant, quadratic, 0.3 mm
Interaction and boundary conditions	Contact/Interaction between bone plate and rollers		Surface-to-surface frictional contact with a COF of 0.1
	Boundary conditions		Supporting rollers are fixed, the loading rollers are only allowed vertical displacement (10 mm)

#### 2.5.2 Sensitivity analysis

In this study, two types of sensitivity analyses were performed depending on the objective. The results were compared and analyzed by setting the QOIs as bending stiffness, yield force ( P ), bending structural stiffness, bending strength, and force at 10 mm displacement in the four-point bending test. Of these, the first four are the performance metrics defined by ASTM F382, whereas the force at 10 mm displacement was included as a supplementary comparison point beyond the standard-defined metrics. The basic relationships required for the QOIs, such as 0.2% offset yield force, bending stiffness, bending structural stiffness, and bending strength in the ASTM F382 four-point static bending test, are described below. Bending stiffness ( K ) was determined via the slope of the linear elastic region in the load-displacement graph ( q=0.002×a , where a is the center span distance). Bending structural stiffness ( $EI_{e}$ ) and bending strength were calculated using Equations 1, 2, respectively:

$$EI_{e}=\frac{\left(2h+3a\right)Kh^{2}}{12}$$ (1)

$$Bending\;strength=\frac{Ph}{2}$$ (2)

where K is the bending stiffness, a is the center span, h is the loading span, and P represents the load at the point where the load-displacement graph intersects the offset curve.

##### 2.5.2.1 Sensitivity analysis of analysis modeling parameters

This analysis was conducted to evaluate the potential sensitivity of simulation results to analysis modeling parameters such as Coefficient of Friction (COF), element type, and element order. This aimed to analyze the dependency of the results on specific settings selected during the analysis process and to verify the appropriateness of the final selected analysis modeling method. The friction coefficient was varied from 0.05 to 0.3 to represent a wide range of friction characteristics. The effects of element type (hexahedral vs. tetrahedral) and element order (linear vs. quadratic) were also varied in separate analyses. For this purpose, four models using Linear Hex, Linear Tets, Quadratic Hex, and Quadratic Tets elements were considered. An average element size of 0.3 mm was used in these simulations.

##### 2.5.2.2 Sensitivity analysis of model input parameters

This sensitivity analysis was performed to identify the extent to which variations in model input values affect model outputs (QOIs). Additionally, the results were used to down-select parameters that do not significantly affect the QOIs, thereby appropriately reducing the scope of the subsequent Uncertainty Quantification (UQ) analysis. The sensitivity analysis evaluated three key model input parameters: elastic modulus ( E ), center span ( a ), and loading span ( h ) as defined in Figure 2D. To quantitatively evaluate the sensitivity of each variable to the QOIs, the local sensitivity coefficient method used in ASME V&V 20 and related prior studies was applied. This method calculates the rate of change in results by applying small perturbations (up/down) to the nominal value of each input parameter. In this study, the perturbation factor α was set to $1\times 10^{-3}$ (i.e., 0.1% of the nominal value) to systematically calculate sensitivity, in contrast to general procedures that vary parameters by arbitrary fixed ratios (e.g., ±10% ). The results are compared as percentage changes relative to the baseline value for three QOIs (bending stiffness, yield force, force at 10 mm).

#### 2.5.3 Uncertainty quantification

Uncertainty Quantification (UQ) helps identify potential limitations caused by inherent variability (aleatoric uncertainty) or lack of knowledge (epistemic uncertainty) in model inputs. This study performed a DOE-based Monte Carlo simulation. The experimental design utilized Latin Hypercube Sampling (LHS) to generate 30 design points. The input parameters selected were elastic modulus ( E ), center span ( a ), and loading span ( h ), which directly affect the calculation formulas for QOIs in the four-point static bending test and the preceding sensitivity analysis (Section 2.5.2.2). The variation range for each parameter was established based on uncertainty evidence: E = 98,500 MPa ( ±1.2% ) referencing prior studies, a = 19 mm ( ±0.2 mm), and h = 16 mm ( ±0.2 mm), assuming a Uniform distribution for all parameters. The sample size was determined by generating LHS designs of n = 10, 20, and 30 and comparing the resulting predictions; the mean predictions of the QOIs agreed across the three designs, and n = 30 was adopted, providing 30 equiprobable strata for each of the three input parameters (Supplementary Appendix SC). For each of the 30 simulations, force-displacement curves were extracted at 0.5 mm increments, and QOIs were calculated from the 30 individual curves by applying the same 0.2% offset procedure described in Section 2.5.2.

#### 2.5.4 Experiment

In this study, a four-point static bending test was performed on a domestic commercial rib bone plate (8-hole) in accordance with the ASTM F382 standard. A total of 5 specimens were prepared. One specimen was excluded from the analysis due to slip at the fixation part during the test; thus, the effective sample size ( n ) was 4. Prior to testing, key dimensions (center span, loading span, etc.) were measured using a caliper. These measurements served as the basis for setting the nominal values for the simulation and the input ranges for UQ described in Section 2.5.3 (e.g., measurement uncertainty, ±0.2 mm). The test was conducted on a setup consisting of two upper loading rollers and two lower support rollers. A load was applied vertically to the specimen via the upper loading rollers at a rate of 3 mm/min (displacement control), and the test continued until the specimen reached 10 mm displacement. Load data were recorded in real-time during the test.

#### 2.5.5 Validation output comparisons

Validation was performed adopting the two-tier comparison approach: a Level I comparison for direct comparison between computational and experimental results using nominal input parameters, and a Level II comparison for quantifying model form error and validation uncertainty based on the mean of the 30 LHS simulations described in Section 2.5.3 (Nagaraja et al., 2024).

##### 2.5.5.1 Level I comparison

In the Level I comparison, QOIs were extracted from bench test data and FEA results and compared. Bending stiffness and yield force (based on 0.2% offset displacement) were calculated using linear regression. A total of 5 QOIs, including directly extracted metrics (bending stiffness, yield force, load at 10 mm displacement) and calculated metrics (bending structural stiffness, bending strength) using geometric data, were averaged from the 4 bench tests. The experimental average QOIs values were compared with the results derived from a FEA simulation using nominal input parameters via an identical calculation procedure, expressed as a percent difference.

##### 2.5.5.2 Level II comparison

In the Level II comparison, model form error and validation uncertainty were quantified. The comparison error ( E ) is defined as the difference between the FEA result ( S ) and the experimental result ( D ), expressed as Equation 3:

E=S−D (3)

The Model Form Error ( $d_{model}$ ) is defined as the comparison error minus the validation uncertainty ( $u_{val}$ ), as defined in Equation 4:

$$d_{model}=E-u_{val}$$ (4)

Validation uncertainty is defined as the combination of numerical uncertainty, input parameter uncertainty, and experimental uncertainty (Equation 5):

$${u_{val}}^{2}={u_{num}}^{2}+{u_{input}}^{2}+{u_{D}}^{2}$$ (5)

Each uncertainty component is constructed as follows: Input parameter uncertainty ( $u_{input}$ ) was derived from the UQ results in Section 2.5.3. The standard deviation of each QOI was calculated from the FEA simulation results for the 30 design points generated via LHS, quantifying the effect of variability in E , a , and h on the output values. Experimental uncertainty ( $u_{D}$ ) includes measurement error and geometric variability between specimens. As explained in Section 2.5.4, this was calculated from the key dimension measurements of the 4 effective samples. In this study, numerical uncertainty ( $u_{num}$ ) was not quantified and was therefore set to $u_{num}=0$ . This is a simplifying assumption: the mesh convergence study established stable convergence behavior at the selected element size (Section 3.1). The implication of this assumption for the reported validation uncertainty is addressed in the Discussion. The Expanded Uncertainty ( $U_{val}$ ) is calculated using a coverage factor k=1.73 , corresponding to a 95% confidence level, as shown in Equation 6:

$$U_{val}=k\times u_{val}$$ (6)

Two metrics were evaluated to determine model acceptability and credibility. First, the normalized error ratio |E|/D indicates whether the model sufficiently predicts experimental results, demonstrating the acceptability of the computational model. The acceptance threshold for this ratio was assigned according to the output comparison gradation of ASME V&V 40-2018, which associates each level with the corresponding level of overall model risk: the Medium level corresponds to differences below 10%, and the High-Medium level to differences below 5% with uncertainty estimated and incorporated into the comparison (ASME, 2018; Supplementary Appendix SA, Supplementary Table SA1). Accordingly, |E|/D < 5% was applied to COU 1 (High-Medium) and |E|/D < 10% to COU 2 (Medium). The validation of the Flat 8H model was evaluated against the 5% threshold, as it provides the evidentiary basis for COU 1. Second, the comparison error (E) was compared with the uncertainty range for the validation judgment. In this study, the expanded uncertainty ( $U_{val}$ ) at a 95% confidence level was used as the final validation criterion, as expressed in Equation 7:

$$\left|E\left|\leq \right.U_{val}\;\left(or\left.\;\right|E\left|/\right.U_{val}\leq 1\right)\right.$$ (7)

This implies that the calculation error ( E ) is completely contained within the 95% confidence interval of the uncertainty range, proving that the model is statistically validated. Conversely, a value of $\left|E\right|/U_{val}>1$ signifies that the calculation error exceeds the uncertainty bounds, suggesting a potential need for model refinement.

## 3 Results

### 3.1 Verification activities

Verification was performed in accordance with the ASME V&V 40 standard, divided into Code Verification and Calculation Verification. This section focuses on the results of Discretization Error Verification, a critical factor determining the numerical accuracy and computational efficiency of the model. Details regarding Code Verification, Numerical Solver Error (NSE), and Use Error are provided separately in Supplementary Appendix SB.

The results of the discretization error verification are presented in Table 4. A convergence study on five mesh sizes (ranging from 1 mm to 0.25 mm) revealed a trade-off where numerical accuracy improved with increased mesh density, but computational time increased exponentially. Consequently, an element size of 0.3 mm (Medium-fine) was selected to balance computational efficiency and accuracy. Comparison with adjacent mesh sizes (0.4 mm and 0.25 mm) showed negligible differences in results, with the maximum equivalent stress changing by 0.71% and −0.57%, and the reaction force changing by −0.33% and 0.00%, respectively.

**TABLE 4:** Results of the discretization error verification.

Mesh size	Change in maximum equivalent stress	Change in reaction force	Change in number of elements	Change in solver time
1 mm (coarse)	1.08%	0.18%	−26.76%	−31.25%
0.5 mm (coarse-medium)	1.32%	−0.03%	−5.59%	−15.79%
0.4 mm (medium)	0.71%	−0.33%	−47.33%	−50.78%
**0.3 mm (medium-fine)**	**−0.57%**	**0.00%**	**−34.25%**	**−47.12%**
0.25 mm (fine)	-	-	-	-

Changes are calculated relative to the next finer mesh size listed in the table. Bold values indicate the selected case.

### 3.2 Validation activities

#### 3.2.1 Results of sensitivity analysis of analysis modeling parameters

To ensure the credibility of the computational model and evaluate the impact of FEA assumptions, we investigated the effects of key modeling parameters, specifically element type, order, and contact conditions (friction coefficient), on the QOIs. Because these modeling parameters were evaluated against experimental averages rather than analytical solutions, this analysis is reported as part of the validation activities. To examine the influence of element type and order on the predicted QOIs, four different element formulations were evaluated: linear tetrahedral, linear hexahedral, quadratic tetrahedral, and quadratic hexahedral (Table 5, Element type and order section). The analysis revealed that element order significantly influenced accuracy. Linear tetrahedral elements overestimated the QOIs by up to 17% compared to experimental averages, while linear hexahedral elements showed deviations of 4%–9%. In contrast, models using quadratic elements demonstrated high accuracy. Both quadratic tetrahedral and quadratic hexahedral elements showed agreement with experimental values within the 5% acceptance threshold, with deviations of approximately 2%–5% across all QOIs. Considering both accuracy and modeling efficiency, quadratic tetrahedral elements were down-selected. Quantifying the deviation associated with each formulation is also intended to inform future four-point bending studies, where geometrically complex rib fixation plates may necessitate a compromise in element selection.

**TABLE 5:** Sensitivity analysis of analysis modeling parameters: normalized comparison of simulation results to experimental average.

Category	Case	Bending stiffness	Yield force	Bending structural stiffness	Bending strength	Force at 10 mm
Element type and order	Experiment (average)	1.00	1.00	1.00	1.00	1.00
	Linear Tets	1.17	1.15	1.17	1.15	1.07
	Linear Hex	1.09	1.07	1.09	1.07	1.04
	**Quadratic Tets**	**1.02**	**1.03**	**1.02**	**1.03**	**0.95**
	Quadratic Hex	1.02	1.03	1.02	1.03	0.95
Coefficient of friction	CoF 0.05	1.02	1.01	1.02	1.01	0.91
	**CoF 0.1**	**1.02**	**1.03**	**1.02**	**1.03**	**0.95**
	CoF 0.15	1.02	1.04	1.02	1.04	0.99
	CoF 0.2	1.03	1.07	1.03	1.07	1.04
	CoF 0.25	1.03	1.09	1.03	1.09	1.08
	CoF 0.3	1.03	1.11	1.03	1.11	1.12

Bold values indicate the selected cases.

To investigate the effect of contact conditions, a sensitivity analysis was performed by varying the COF between the bone plate and rollers from 0.05 to 0.3 (Table 5, Coefficient of friction section). The results indicated a trend where QOIs gradually increased with the COF. Deviations from experimental values increased as the COF exceeded 0.15, with yield force and bending strength predicted to be 11% higher than experimental averages at a COF of 0.3. The force at 10 mm displacement was particularly sensitive to changes in COF, decreasing by 9% at a COF of 0.05 and increasing by 12% at 0.3. A COF of 0.1 yielded results closest to the experimental values (QOI deviation of 2%–5%) and was therefore adopted as the baseline setting.

#### 3.2.2 Results of sensitivity analysis of model input parameters

A sensitivity analysis was conducted to quantify the impact of key model input parameters, elastic modulus (E), center span (a), and loading span (h), on the QOIs, applying a perturbation of ±0.1% to the nominal value of each parameter. The results are presented in Figure 3. The analysis indicated that the loading span (h) had the most dominant effect on the QOIs across all three response variables. For the bending stiffness, the loading span (h) showed the highest sensitivity with a change rate of approximately 0.25%, followed by the elastic modulus (E) at approximately 0.10% and the center span (a) at approximately 0.07%. The sensitivity of h was approximately 2.5 times higher than that of E and 3.6 times higher than that of a. The same dominance of h was also observed for the yield force and the force at 10 mm displacement, with maximum change rates of approximately 0.15% and 0.16%, respectively, confirming that h is the most influential parameter regardless of the QOI considered. The sensitivity order for the bending stiffness was identified as h > E > a. This suggests that the results of the four-point bending test are more sensitive to the geometric precision of the test setup (h) than to the material properties (E). Based on these results, all three parameters were considered key input variables for the subsequent UQ analysis (Section 3.2.3).

**FIGURE 3:**
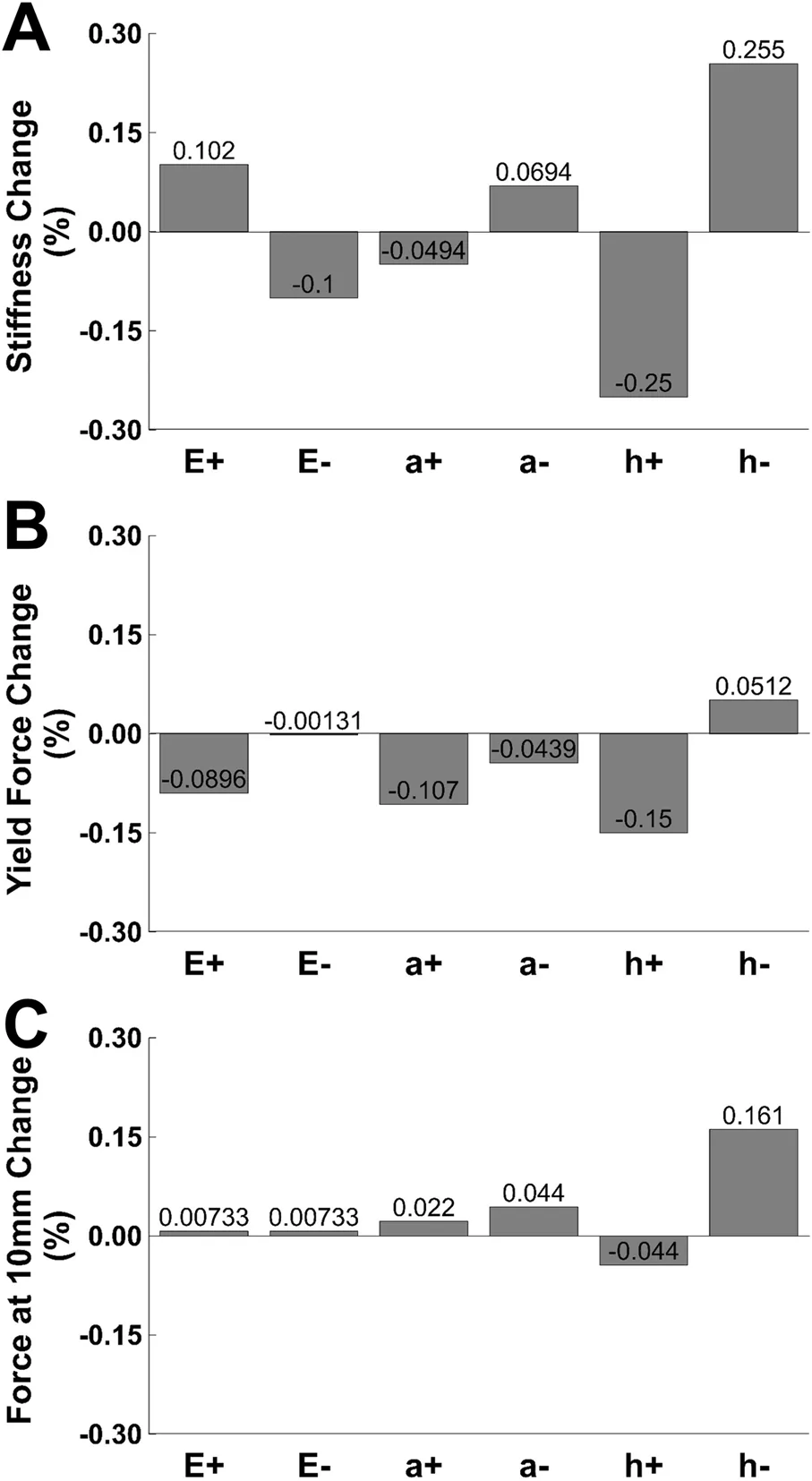
Results of sensitivity analysis for model input parameters. Percent change in **(A)** bending stiffness, **(B)** yield force, and **(C)** force at 10 mm displacement relative to variations in elastic modulus ( E ), center span ( a ), and loading span ( h ).

#### 3.2.3 Uncertainty quantification

To quantify the input uncertainty ( $u_{input}$ ) of the simulation results caused by variations in the key input parameters ( E,a,h ) identified in the sensitivity analysis (Section 3.2.2), 30 design points were generated using LHS, and FEA was performed. The input uncertainty ( $u_{input}$ ) was calculated as the standard deviation of the results from the 30 simulations and analyzed in detail along the entire load-displacement curve (0.5 mm–10 mm) (Figure 4). The analysis showed that the maximum input uncertainty (standard deviation) was observed at 1.90 N at a displacement of 2 mm. At displacements of 0.5 mm and the final 10 mm, the uncertainties were 0.54 N and 0.88 N, respectively. The mean predictions of the QOIs agreed across the LHS designs of n = 10, 20, and 30, with the 95% confidence intervals of the means sharing a common interval, and n = 30 was adopted for the values reported here (Supplementary Appendix SC, Supplementary Figure SC1).

**FIGURE 4:**
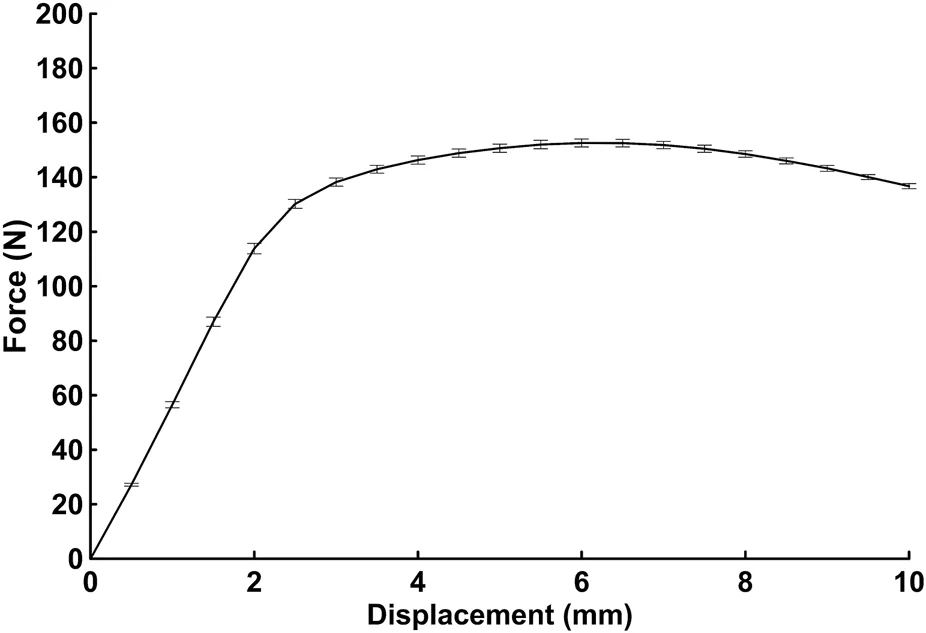
Uncertainty Quantification results. The graph depicts the uncertainty in the FEA results represented as force-displacement curves.

#### 3.2.4 Experimental comparator

Prior to testing, the key geometric dimensions of the specimens were measured using a caliper. The measurement results indicated a center span ( a ) of 19.0±0.2 mm and a loading span ( h ) of 16.0±0.2 mm. The measured standard deviation ( ±0.2 mm) was used to define the variability of the input parameters.

### 3.3 Equivalency comparison

#### 3.3.1 Level I equivalency comparison

Table 6 summarizes the experimental data from the four-point static bending test and the FEA results. Figures 5A–C displays the load-displacement curves of the FEA results alongside the 95% confidence intervals of the experimental data. In Level I comparison, errors for all QOIs remained within 5% (Table 6). Qualitatively, the load-displacement curves showed close agreement in the initial elastic region, although some discrepancies were observed in the post-yield plastic region. The FEA data were considered to be within an acceptable range relative to the 95% confidence interval of the experimental data.

**TABLE 6:** Comparison of quantities of interest between experimental testing and computational modeling.

Method	Bending stiffness (N/mm)	Yield force (N)	Bending structural stiffness (Nmm^2^)	Bending strength (Nmm)	Force at 10 mm (N)
Experiment (average)	57.65	111.01	109452.17	888.04	143.28
CM&S	58.93	113.88	111888.43	911.06	136.46

**FIGURE 5:**
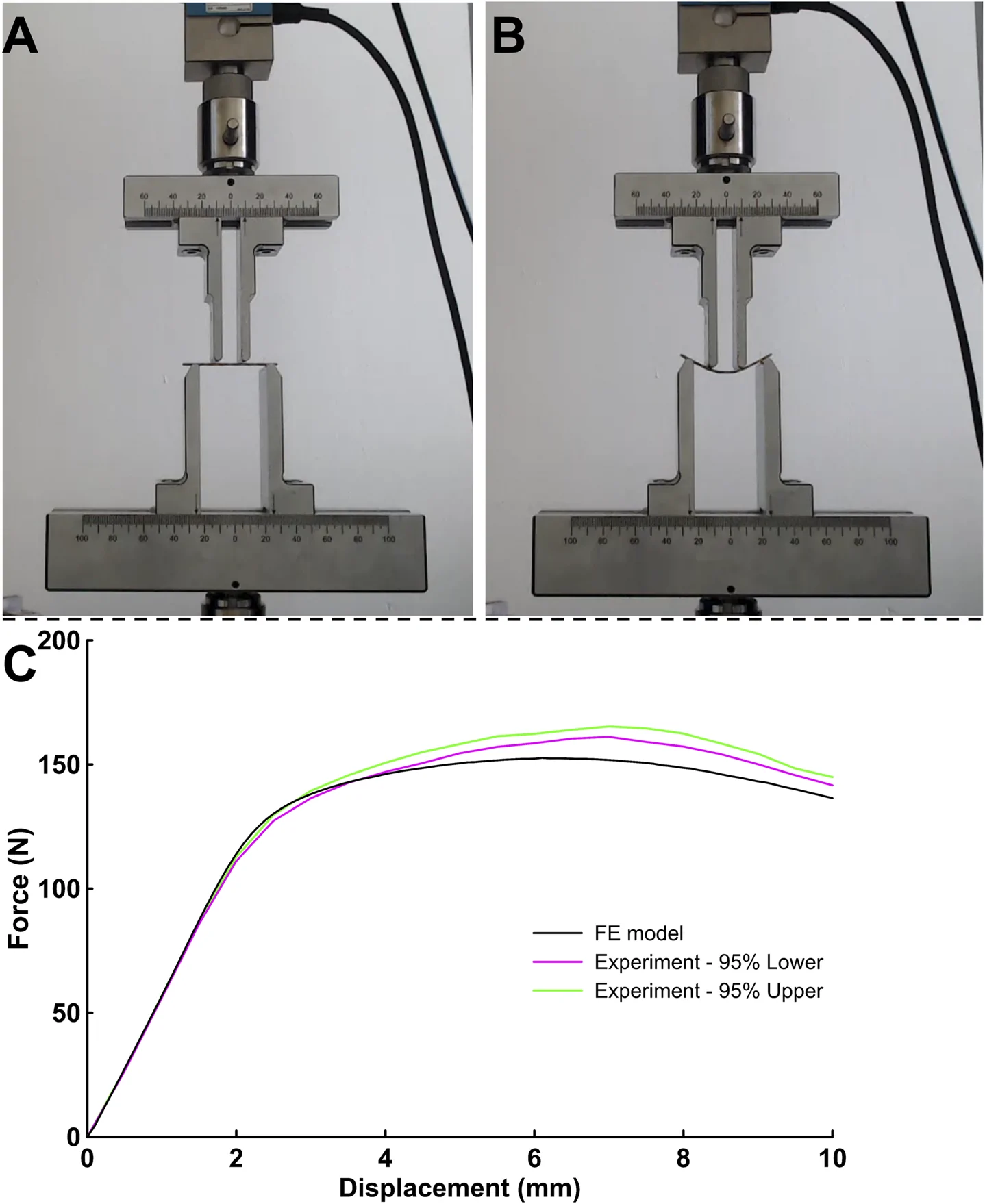
Validation of the FE model against experimental bench testing. **(A)** Initial experimental setup prior to loading. **(B)** Deformed shape of the specimen under load. **(C)** Comparison of the computational force-displacement curve (black line) with the experimental results (colored lines representing 95% confidence intervals).

#### 3.3.2 Level II equivalency comparison

The mean values and uncertainties for the QOIs are summarized in Table 7, alongside validation metrics ( E , $u_{val}$ , and $U_{val}$ ) in the experimental (Expt.) and simulation (FEA) columns. The ratio of comparison error ( E ) to measurement value ( D ), denoted as E/D , was calculated to be 2.19% for bending stiffness, 2.84% for yield force, 2.18% for bending structural stiffness, and 2.84% for bending strength, with the largest difference observed in the force at 10 mm displacement at −4.74%. Consistent with the Level I analysis, the E/D values for all QOIs fell within the target error rate of 5%.

**TABLE 7:** Validation metrics and uncertainty analysis results for the quantities of interest.

QOIs	Experiment		FE model		Comparison error	Total uncertainty	Expanded uncertainty
	D	uD	S	$u_{input}$	E=S−D	$u_{val}={\left(u_{D}^{2}+u_{input}^{2}\right)}^{1/2}$	$U_{val}=1.73\times u_{val}$
Bending stiffness (N/mm)	57.65	0.19	58.90	1.04	1.26	1.06	1.83
Yield force (N)	111.01	1.54	114.15	1.44	3.15	2.11	3.64
Bending structural stiffness (Nmm^2^)	109452.17	366.03	111839.98	1970.16	2387.81	2003.87	3466.70
Bending strength (Nmm)	888.04	4.50	913.23	11.52	25.18	12.37	21.39
Force at 10 mm (N)	143.28	1.51	136.49	0.88	−6.79	1.75	3.02

Experimental result ( D ), experimental uncertainty ( uD ), FEA result ( S ), input parameter uncertainty ( $u_{input}$ ), comparison error ( E ), validation uncertainty ( $u_{val}$ ), and expanded uncertainty ( $U_{val}$ ) are defined in Section 2.5.5.2.

We conservatively evaluated the model validity by analyzing the comparison error ( E ) based on the expanded uncertainty at a 95% confidence level ( $U_{val}=1.73\times u_{val}$ ). The analysis results showed that $\left|E\left|/\right.U_{val}\right.$ was less than 1 for key mechanical properties such as bending stiffness (0.689), yield force (0.865), and bending structural stiffness (0.689). This implies that the calculation error ( E ) for these QOIs lies within the expanded uncertainty ( $U_{val}$ ) range even under the 95% confidence criterion, supporting model adequacy under the stated Context of Use and the uncertainty model considered in this study. While items such as bending strength (1.177) and force at 10 mm displacement (2.248) showed $\left|E\left|/\right.U_{val}\right.$ values exceeding 1, this indicates that the calculation error ( E ) for these items is larger than the noise band of uncertainty ( $U_{val}$ ) and is thus quantifiable. However, as confirmed in the E/D analysis, the absolute error rates for these items (2.84% and −4.74%, respectively) are within the acceptable range of less than 5%, so the overall predictive performance of the model is judged to be acceptable.

This trend is also evident in the displacement-dependent analysis shown in Figure 6. The $E/D\left(\%\right)$ graph showed negative errors in the plastic region after the yield point (approximately 2 mm), with differences reaching up to approximately 7%, exceeding the 5% criterion. The rapid decrease in the $E/U_{val}$ graph in the plastic region can be interpreted as an indication that the actual calculation error ( E ) is becoming significantly larger than the expanded uncertainty ( $U_{val}$ ) due to the limitations of the applied bilinear material model.

**FIGURE 6:**
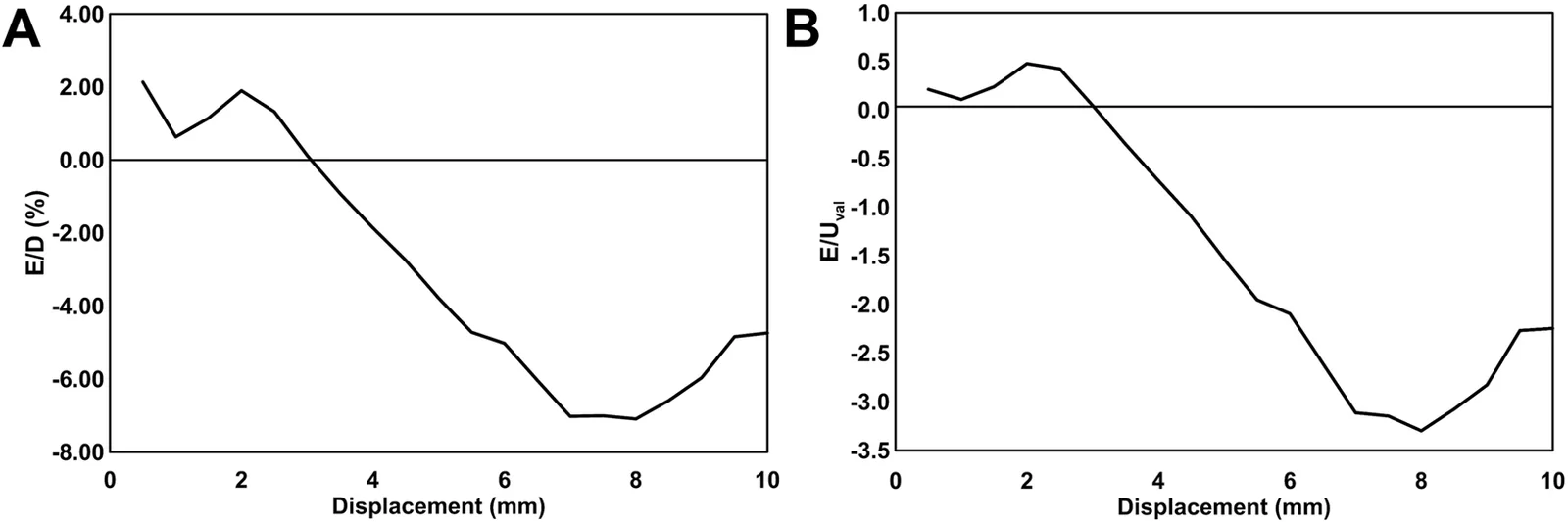
Quantitative validation metrics plotted against displacement. **(A)** Relative comparison error percentage ( E/D ). **(B)** Ratio of comparison error to expanded uncertainty ( $E/U_{val}$ ).

### 3.4 COU 1 (QOI 1): design change (increased hole count)

To evaluate the effect of increasing the number of holes on the mechanical performance of rib bone plates, modeling and simulations were performed for 10-hole and 12-hole plates based on the Flat 8H model validated in Section 3.3 (Figure 7A). All simulations adhered to the test configuration of the ASTM F382 standard. This standard defines the center span ( a ) and loading span ( h ) based on the number of local screw holes between rollers (two each) rather than the total length of the plate (F382, 1.6.1.3.1 and 1.6.1.3.2). Therefore, in QOI 1, increasing the hole count effectively changes only the total length of the plate. The same center span ( a ) and loading span ( h ) were thus applied to all three models (8H, 10H, 12H). All other analysis conditions, including load conditions, boundary conditions, and material properties, were maintained identical to the validated 8-hole model.

**FIGURE 7:**
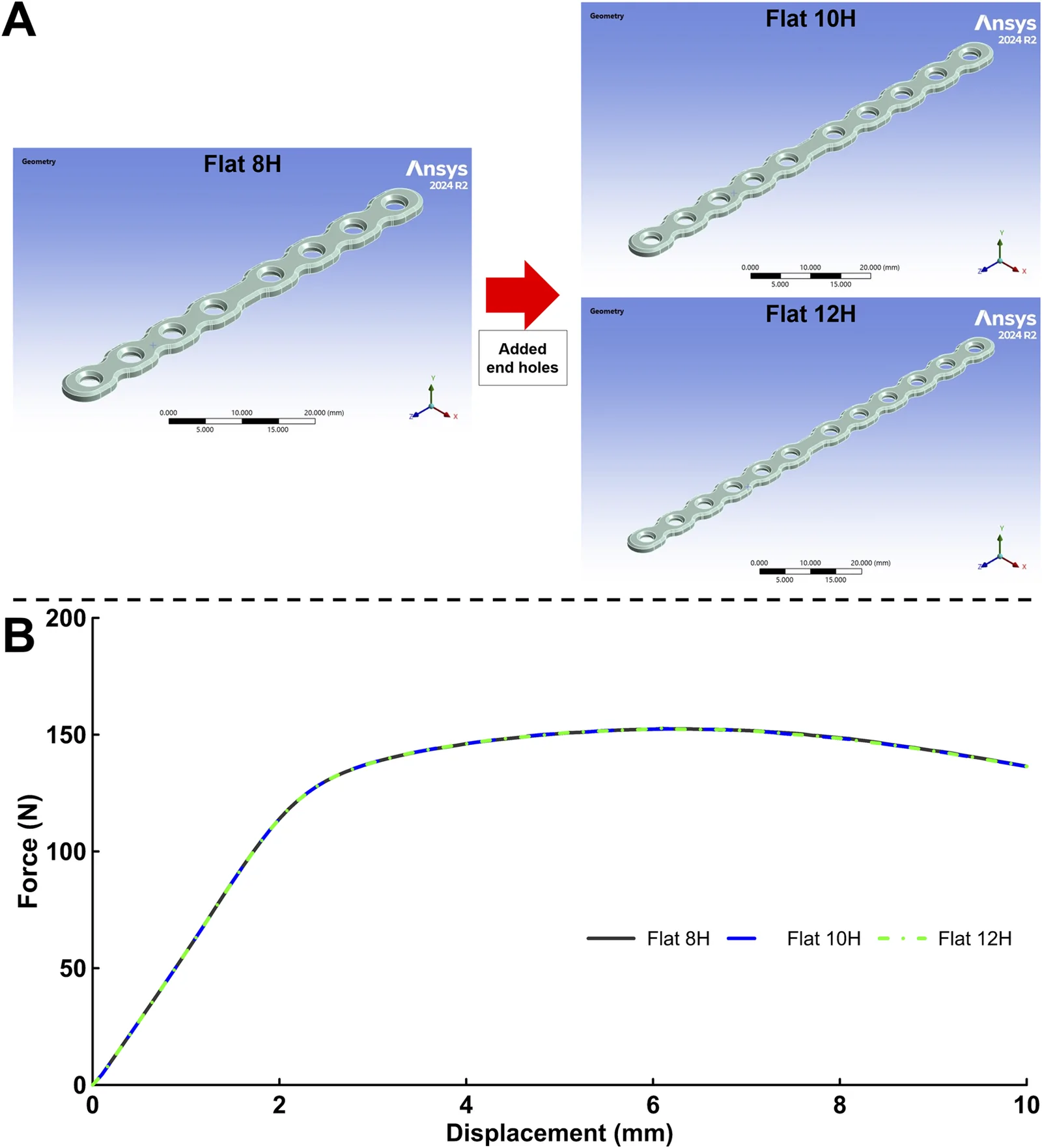
Evaluation of COU 1: Impact of increasing hole count on mechanical performance. **(A)** Finite element model configurations for the baseline Flat 8-hole (8H) plate and the extended Flat 10-hole (10H) and 12-hole (12H) designs. **(B)** Predicted force-displacement curves for the three designs.

Table 8 summarizes the comparison results of key QOIs for the three designs. Looking at the results normalized to the 8-hole model, the changes in bending stiffness, bending structural stiffness, and force at 10 mm displacement with increasing hole count were within 0.05%, indicating virtually no change. Slight increases of less than 0.2% were observed only in yield force and bending strength, but these are not at a level that significantly affects mechanical performance. The comparison of load-displacement curves also indicates that there is no appreciable difference in mechanical behavior among the three models (8H, 10H, 12H), as the curves virtually overlap across the entire elastic and plastic regions (Figure 7B). Based on the simulation results, it is predicted that when a four-point bending test with fixed spans ( a,h ) is performed according to the ASTM F382 standard (1.6.1.3), key mechanical performance metrics such as bending stiffness or yield force would not degrade even if the total number of holes (i.e., length) of the plate increases. This model-predicted trend is consistent with the regulation that plates of different lengths with the same nominal cross-section can be considered as a single sample (F382, 1.7.2).

**TABLE 8:** Comparison of mechanical parameters across plate design variations evaluated in COU 1 and COU 2.

COU	Design	Method	Stiffness (N/mm)	Yield force (N)	Bending structural stiffness (Nmm^2^)	Bending strength (Nmm)	Force at 10 mm (N)
COU 1 (Hole count)	Flat 8H	Experiment	57.65 ± 0.25	111.01 ± 1.06	109452.17 ± 465.84	888.04 ± 8.47	143.28 ± 0.88
		FE model	58.93	113.88	111888.43	911.07	136.46
	Flat 10H	FE model	58.93	113.98	111890.15	911.83	136.38
	Flat 12H		58.91	114.04	111845.28	912.30	136.44
COU 2 (pre-contoured)	Experiment	Concave 8H	66.59 ± 4.33	92.68 ± 6.19	135961 ± 8837.89	764.59 ± 51.05	99.82 ± 7.37
			71.15	98.49	145279.41	812.51	102.94
	FE model	Convex 8H	45.71 ± 2.35	101.85 ± 9.73	93333.64 ± 4803.04	840.3 ± 80.25	176.97 ± 2.80
			46.27	108.95	94479.82	898.8	180.63

### 3.5 COU 2 (QOI 2): product line extension (pre-contoured shapes)

This section evaluates the QOI of COU 2, specifically whether the validated Flat 8H model can be extended to pre-contoured shapes. Two geometric conditions, convex (Convex 8H) and concave (Concave 8H), were defined by the orientation in which the pre-contoured plate was mounted. Five specimens were tested in each orientation, giving ten specimens in total, under the same loading configuration used for the Flat 8H validation. In accordance with the definition of COU 2, simulations were performed for these two conditions based on the framework of the validated Flat 8H model (Figure 8E). Load conditions, boundary conditions, material properties, and mesh settings were applied identically to the validated model. Figures 8A–D shows the states of the Convex 8H (A, B) and Concave 8H (C, D) specimens before and after the bench test.

**FIGURE 8:**
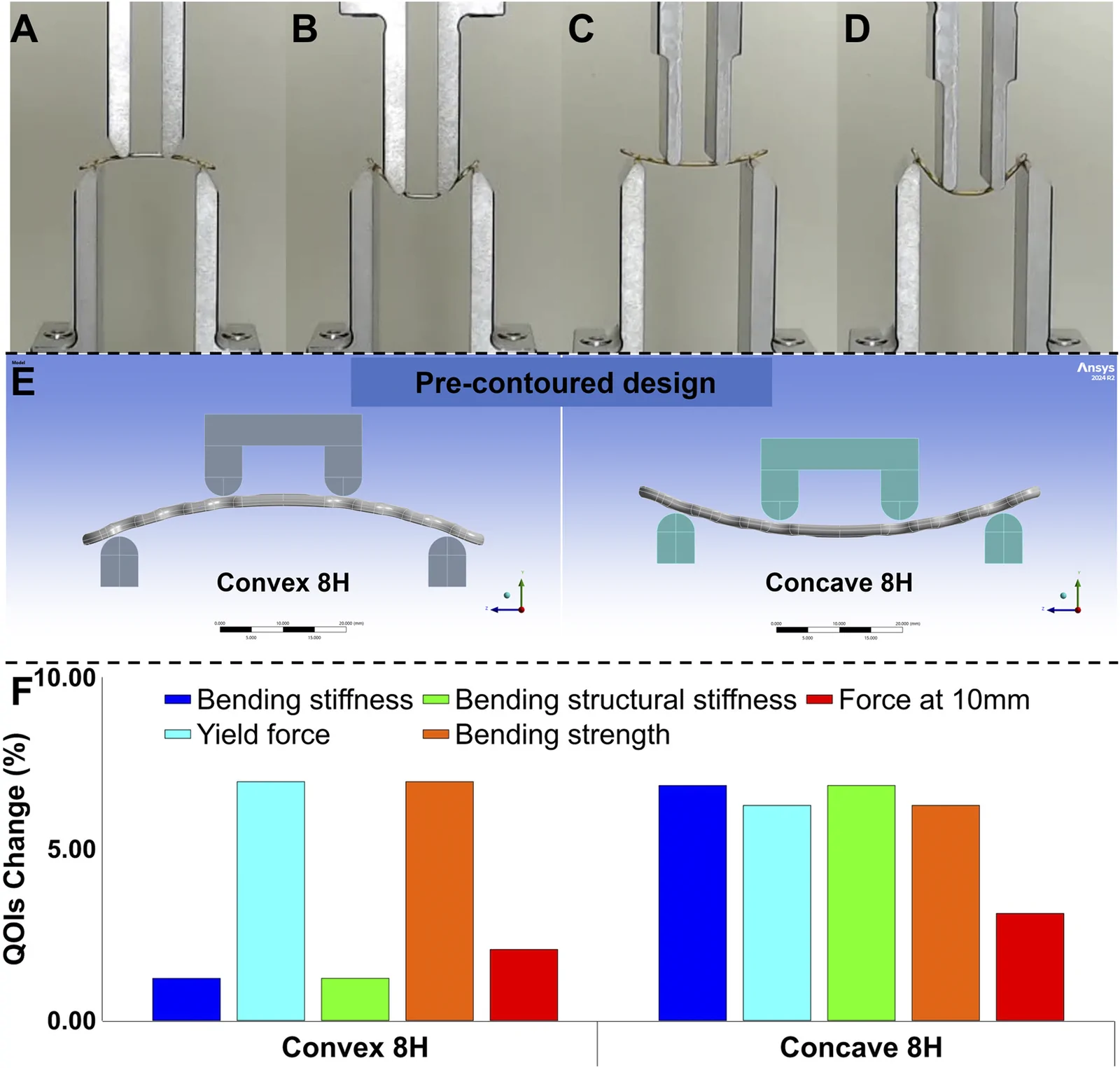
Evaluation of COU 2: Applicability of the model to pre-contoured shapes. **(A–D)** Experimental configurations showing pre- and post-test states for Convex 8H **(A,B)** and Concave 8H **(C,D)** plates. **(E)** Finite element model setups corresponding to the convex and concave geometries. **(F)** Comparison of the percentage prediction error of the simulation for Quantities of Interest (QOIs) against experimental results.

As defined in the COU, the CM&S results were cross-compared and validated against the bench test results. Table 8 summarizes the experimental and simulation results for Convex 8H and Concave 8H, while Figure 8F presents the percentage error of the simulation results relative to the experimental values. The analysis revealed that simulation errors were within the 10% threshold established for COU 2 for all QOIs, including bending stiffness and yield force, for both Concave 8H and Convex 8H models. This confirms that the CM&S model reliably predicts mechanical performance according to pre-contouring and positioning orientation within the same 8-hole product family, indicating that the model is valid as supplementary evidence (Medium Model Influence) as defined in COU 2.

## 4 Discussion

This study performed a credibility assessment based on the ASME V&V 40 standard to evaluate the mechanical performance of rib bone plates. While recent end-to-end V&V case studies have established credibility protocols for spinal implants (Nagaraja et al., 2020; 2024), these prior efforts have largely focused on a single Context of Use. This study extends these foundational efforts by demonstrating how credibility activities can be scaled and contrasted across two distinct Contexts of Use within a single coherent framework, providing practical insights into how the same validated model can serve regulatory decisions of differing risk levels. Specifically, by presenting COU 1 (utilizing CM&S as stand-alone evidence) and COU 2 (utilizing CM&S as supplementary evidence) side by side, we illustrate how VVUQ activities can be tailored to differing model risks (High–Medium and Medium–Medium), illustrating how CM&S may be utilized in the regulatory approval of Class III trauma devices (MFDS, 2025). It should be noted that the credibility demonstrated in this study is confined to titanium rib bone plates, the ASTM F382 four-point bending loading configuration, and geometries similar to those included in the validated plate family. Although the discussion below addresses the broader implications of these findings for risk-informed V&V practice, the credibility claims themselves are to be understood within this specific material, loading condition, and design space.

In the verification activities, the NCV error rate was found to be low at 0.704% for the maximum normal stress, and discretization error verification confirmed stable convergence at the selected element size of 0.3 mm, with deviations from adjacent mesh sizes remaining below 1%. These results suggest that the numerical accuracy and stability of the computational model were adequate for the subsequent validation activities. In particular, the sensitivity analysis of analysis modeling parameters revealed that while linear elements overestimated stiffness by up to 17%, quadratic tetrahedral elements demonstrated high accuracy. This finding aligns with the fundamental study (Cifuentes and Kalbag, 1992), which demonstrated that quadratic tetrahedral elements achieve close agreement with analytical solutions in bending-dominant problems, whereas linear tetrahedra can introduce significant errors. Furthermore, explicitly justifying the element order addresses the reporting standards emphasized by (Baumann et al., 2021), who highlighted that rigorous reporting of mesh characteristics is critical for the regulatory assessment of orthopedic implants.

The results showing error rates ( E/D ) of less than approximately 2.9% for bending stiffness and yield force in the Level I comparison of validation activities imply that the computational model accurately predicts the initial mechanical behavior of the rib bone plate within the elastic region. More importantly, for these key QOIs, the calculation error ( E ) was contained within the expanded uncertainty ( $U_{val}$ ) range at a 95% confidence level ( $\left|E\left|/\right.U_{val}<1\right.$ ), supporting model adequacy under the stated Context of Use and the uncertainty model considered in this study. Because the 5% threshold applied to E/D corresponds to the rigor level appropriate to the assessed model risk under ASME V&V 40-2018 rather than to a universally applicable value, and because deviations of this magnitude do not alter the assessment of whether the plate meets the mechanical performance requirements of ASTM F382, the criterion provides assurance that model error remains small enough not to affect conclusions regarding device performance.

This indicates that the material properties (elastic modulus) and geometric implementation applied to the model appropriately simulate the actual physical phenomena, supporting the credibility of the model within the established COU. The selection of these QOIs was not merely compliant with ASTM standards but was also driven by their direct correlation with clinical failure modes. For instance, reliably predicting bending stiffness and yield strength is critical for preventing hardware failure during the physiological loading of breathing and coughing (Bottlang et al., 2010), as well as ensuring adequate mechanical stability to facilitate bone healing.

The results showing that the error rate ( E/D ) increased to a maximum of approximately −7% in the plastic region, and that the value of $\left|E\left|/\right.U_{val}\right.$ exceeded 1 specifically for bending strength and force at 10 mm displacement, suggest that model form error exists and that its cause is quantifiable rather than being obscured by uncertainty noise. The reasons why these two QOIs did not meet the validation criterion ( $\left|E\left|/\right.U_{val}\leq 1\right.$ ) are analyzed differently. First, the force at 10 mm displacement is located in a region where significant plastic deformation occurs after the material passes the yield point. Therefore, the result of $\left|E\left|/\right.U_{val}>1\right.$ for this metric is attributed to simplified representation of the strain hardening behavior of Titanium Grade 4 by the bilinear material model, which was adopted because the performance metrics defined by ASTM F382 are governed by the elastic response and the yield point rather than by post-yield hardening. This limitation is consistent with FDA lab data from a multi-center spinal rod benchmark (Nagaraja et al., 2020). Their results showed an error of 0.7% in the elastic region, which increased to 4.6% in the full post-yield regime. This comparison indicates that the reduced accuracy in the plastic zone is a recognized consequence of simplified material modeling rather than a deficiency in the validation process. On the other hand, the exceedance observed for bending strength arises from the way the metric is constructed rather than from the predictive behavior of the model. Bending strength is calculated using Equation 2 ( $\left.Ph/2\right)$ , so with a loading span of h = 16 mm the comparison error is amplified eightfold relative to that of the yield force, whereas the validation uncertainty is not amplified to the same degree. The ratio $\left|E\left|/\right.U_{val}\right.$ for bending strength is therefore larger than that for the yield force, and the observed deviation should be interpreted in the context of this characteristic of the performance metric itself. From the perspective of the ASME V&V standard, although the error rates ( E/D ) for these QOIs were within or close to the acceptable range (less than 5%), the result of $\left|E\left|/\right.U_{val}>1\right.$ implies that there is room to further improve prediction accuracy through future refinement of the CM&S FE model.

In COU 1 (QOI 1), the finding that increasing the number of holes (10H, 12H) in the rib bone plate had an impact of less than 0.2% on mechanical performance suggests that changes in plate length do not affect bending performance under the ASTM F382 standard. This is because the test standard defines the span based on the local region between rollers rather than the total length of the plate. To derive this result, this study performed V&V activities commensurate with the High-Medium model risk assessed for this COU and demonstrated model credibility within the validated domain. It should be emphasized, however, that this conclusion rests on CM&S extrapolation from the validated Flat 8H model, as no physical bench testing was performed for the 10-hole and 12-hole configurations. These results therefore represent a model-predicted trend rather than experimentally confirmed equivalence. Experimental confirmation of the 10-hole and 12-hole configurations would provide an additional layer of evidence to complement the present model-based findings, and would be required before CM&S could serve as stand-alone evidence for regulatory decision-making on such design changes.

In COU 2 (QOI 2), the result showing a prediction error of less than 10% (maximum 7%) when extending the validated model to pre-contoured shapes suggests that the developed CM&S framework provides credible prediction performance for changes in shape and positioning orientation within the same 8-hole product family, as defined in the current COU. This level of agreement is consistent with the role of supplementary evidence associated with the Medium-Medium model risk. However, it must be clarified that the applicability of this model is limited to the current validation domain. The current results demonstrate prediction performance for bending shape variations within a range that shares the basic 8-hole design. If the model is extrapolated to broader design changes outside the COU, such as significantly different hole counts (e.g., 12 holes or more) or complex cross-sectional geometries, prediction uncertainty may increase. According to the ASME V&V 40 standard, additional validation activities covering the new design space are required to expand the model applicability. Nevertheless, these results show that when CM&S is used complementarily with physical testing within the validated range, it can support regulatory requirements while significantly improving the development efficiency of orthopedic implant product families.

A design choice of this study was to apply a single credibility framework, calibrated to the higher-risk COU (High-Medium), to both COUs rather than constructing two separate frameworks. The activities performed under this framework satisfied the credibility requirements for COU 1 by design. Since the hierarchical grade structure of ASME V&V 40 means that High-Medium inherently exceeds Medium-Medium, the same outcomes satisfy the requirements for COU 2 as well. According to the output comparison gradation of ASME V&V 40-2018 (ASME, 2018; Supplementary Appendix SA, Supplementary Table SA1), each overall model risk level is associated with a required level of output comparison, and the Medium level corresponds to comparing the arithmetic difference alone, at a 10% threshold. Neither model nor comparator uncertainty is incorporated at this level, and it corresponds to the Level I comparison. Uncertainty is introduced only at the High-Medium level required for COU 1. Had COU 2 been addressed on its own, the uncertainty-related factors, including Rigor of Output Comparison, Model Inputs: Quantification of Uncertainties, and the associated measurement-uncertainty factors, could therefore have been graded lower. This conservative envelope approach may be useful where multiple COUs share overlapping model behaviors but differ in risk level.

This study has several limitations. First, as discussed previously, the use of a bilinear material model reduced prediction accuracy in the plastic region. The performance metrics defined by ASTM F382 are determined within the elastic region or at the yield point, and the reduced accuracy in the plastic region therefore had a limited impact on the main validation conclusions of the model. A nonlinear hardening representation would be expected to improve agreement in the post-yield region, where the deviation of the force at 10 mm displacement arises, but would not be expected to materially alter the F382-defined metrics, which are governed by the elastic response and the yield point. Extending the model to a context of use in which the post-yield response is itself the quantity of interest would require a nonlinear material representation and a corresponding tensile characterization of the specimen material. Second, numerical uncertainty was not quantified in this study and was assumed to be zero in Equation 5. The mesh convergence study established stable convergence behavior, and the element size of 0.3 mm was selected as the balance between accuracy and computational cost, with deviations from adjacent mesh sizes remaining below 1% (Table 4). This verification does not, however, replace a rigorous quantification of numerical uncertainty through established methods such as the Grid Convergence Index (GCI) or Richardson extrapolation. As a consequence, the validation uncertainty reported in this study may be underestimated, and the comparison between computational and experimental results should be interpreted with this in mind. This assumption is consistent with the credibility grade assigned to the “Discretization Error” factor (level b), which reflects that convergence behavior was observed to be stable but the discretization error itself was not estimated. A comprehensive quantification of numerical uncertainty is identified as an important area for future refinement of the present framework. Third, the thread geometry was simplified for computational efficiency. This simplification may limit the extrapolation of the model to analyses of stress concentration or complex contact mechanisms in actual clinical situations. Fourth, the experiment was conducted at a single institution with a limited number of specimens (n = 4) per test condition, which constrains the statistical power of the validation and limits the characterization of experimental variability. This sample size is insufficient to capture inter-laboratory variability or to demonstrate modeling reproducibility, and it contrasts with prior studies (Nagaraja et al., 2020; 2024) that demonstrated model robustness through multi-center collaboration. This constraint was reflected explicitly within the credibility framework rather than left unaddressed: in accordance with ASME V&V 40-2018, the credibility factors “Quantity of Test Samples” and “Quantity of Test Conditions” were assigned lower gradations (levels b and a, respectively), so that the credibility goals were moderated in proportion to the available evidence. Nevertheless, although the physical testing was limited to a single loading condition, the evaluation scope of the computational model extended to bending shape variations. Extending the utility of baseline experimental data (*n* per condition, single institution) to evaluate diverse product configurations was a key practical motivation for developing a credible CM&S framework. Indeed, the result that the model validated in COU 2 (QOI 2) provided credible predictions (error within 10%) across these various conditions (pre-contoured shapes, Convex/Concave) suggests that the CM&S framework developed in this study can serve as an effective evaluation tool for geometric variations within its validated range.

Future research should prioritize conducting multi-center studies to validate reproducibility. Notwithstanding these limitations, this study presents a preliminary framework for assessing the credibility of CM&S models for orthopedic implants. Given the limited sample size (n = 4) and the single-institution experimental design, the conclusions regarding model credibility and regulatory applicability should be considered preliminary at this stage, and further validation with larger datasets and multi-center testing would provide a stronger evidentiary basis. Nevertheless, the risk-informed approach demonstrated here may offer methodological insights beyond rib bone plates, potentially informing future credibility assessments for patient-specific implants (PSI) and additively manufactured devices where traditional physical testing is often impractical. These broader applications remain to be investigated and should not be regarded as established conclusions of the present work.

## Data Availability

The original contributions presented in the study are included in the article/Supplementary Material, further inquiries can be directed to the corresponding author.
