# Supplementary material for: Risk-informed credibility goals for finite element simulation of rib bone plates: a case study in applying ASME V&V 40 to ASTM F382 testing
Source: Front Bioeng Biotechnol. 2026 Oct 26;14:1875854. doi: 10.3389/fbioe.2026.1875854 (PMC13745701; doi:10.3389/fbioe.2026.1875854)
Supplement: Supplementary file 1 [file Supplementaryfile1.docx]

Supplementary Material

# APPENDIX A

Table SA1 presents the validation metric corresponding to each level of overall model risk, on which the acceptance criteria applied in this study were based. The credibility factors defined in ASME V&V 40 are listed in Tables SA2 through SA4. In each table, the credibility factors applicable to the current simulation are highlighted in bold.

**Table SA1.** Validation metric for each level of overall model risk, based on the output comparison gradation of ASME V&V 40-2018 (ASME, 2018)

| Level | Description |
| --- | --- |
| Low | Visual comparison concludes good agreement. |
| Low-Medium | Comparison by measuring the difference between computational results and experimental data. Differences are less than 20%. |
| Medium | Comparison by measuring the difference between computational results and experimental data. Differences are less than 10%. |
| High-Medium | Comparison with uncertainty estimated and incorporated from the comparator or computational model. Differences between computational results and experimental data are less than 5%. Includes consideration of some uncertainty, but statistical distributions for uncertainty quantification are unknown. |
| High | Comparison with uncertainties estimated and incorporated from both the comparator and the computational model, including comparison error. Differences between computational results and experimental data are less than 5%. Statistical distributions for uncertainty quantifications are known. |

**Table SA2.** Goals for credibility factors associated with Verification

| Code Verification | Software Quality Assurance (SQA)  a. Very little or no SQA procedures were specified or followed.  b. SQA procedures were specified and documented.  c. SQA procedures were specified and documented; the software anomaly list and the software development environment are fully understood and the impact on the COU is analyzed and documented; quality metrics are tracked.  Numerical Code Verification (NCV)  a. NCV was not performed.  b. The numerical solution was compared to an accurate benchmark solution from another verified code.  c. Discretization error was quantified by comparison to an exact solution, and a grid convergence study demonstrated that the numerical solution asymptotically approached the exact solution as the discretization was refined.  d. In addition to (c), the observed order of accuracy was quantified and compared to the theoretical order of accuracy. |
| --- | --- |
| Calculation Verification | **Discretization Error**  a. No grid convergence performed.  **b. Applicable grid convergence analyses were performed and their respective convergence behaviors were observed to be stable, but the discretization error was not estimated.**  c. Applicable grid convergence analyses were performed and discretization error was estimated. |
|  | **Numerical Solver Error (NSE)**  a. No solver parameter sensitivity was performed.  b. No solver parameter sensitivity was performed. Solver parameters were established based on values from a previously verified computational model.  **c. Problem-specific sensitivity study was performed on solver parameters, confirming that changes in simulation results due to changes in the solver parameters were negligible relative to the model accuracy goal.** |
|  | **Use Error**  a. Inputs and outputs were not verified.  b. Key inputs and outputs were verified by the practitioner.  **c. Key inputs and outputs were verified by internal peer review.**  d. Key inputs and outputs were verified by reproducing simulations as part of an external peer review. |

**Table SA3.** Goals for Credibility Factors Associated with Validation

| Computational Model | Model Form  a. Influence of model form assumptions was not explored.  b. Influence of expected key model form assumptions was explored.  c. Comprehensive evaluation of model form assumptions was conducted.  Model Inputs: Quantification of Sensitivities  a. Sensitivity analysis was not performed.  b. Sensitivity analysis on expected key parameters was performed.  c. Comprehensive sensitivity analysis was performed.  Model Inputs: Quantification of Uncertainties  a. Uncertainties were not identified.  b. Uncertainties on expected key inputs were identified and quantified, but were not propagated to quantitatively assess the effect on the simulation results.  c. Uncertainties on all inputs were identified and quantified, and were propagated to quantitatively assess the effect on the simulation results. |
| --- | --- |
| Comparator | **Quantity of Test Samples**  a. A single sample was used.  **b. Multiple samples were used, but not enough to be statistically relevant.**  c. A statistically relevant number of samples were used.  **Range of Characteristics of Test Samples**  a. One or more samples with a single set of characteristics were included.  **b. Samples representing a range of characteristics near nominal were included.**  c. Samples representing the expected extreme values of the parameters were included.  d. Samples representing the entire range of parameters were included.  **Measurements of Test Samples**  a. Test samples were not measured and/or characterized.  **b. One or more key characteristics of the test samples were measured.**  c. All key characteristics of the test samples were measured.  **Uncertainty of Test Sample Measurements**  a. Samples were not characterized or were characterized with gross observations, and measurement uncertainty was not addressed.  b. Uncertainty analysis incorporated instrument accuracy only  **c. Uncertainty analysis incorporated instrument accuracy and repeatability (i.e., statistical treatment of repeated measurements).**  d. Uncertainty analysis incorporated a comprehensive uncertainty quantification, which included instrument accuracy, repeatability, and other conditions affecting the measurements.  **Quantity of Test Conditions**  **a. A single test condition was examined.**  b. Multiple (two to four) test conditions were examined.  c. More than four test conditions were examined.  **Range of Test Conditions**  **a. A single test condition was examined.**  b. Test conditions representing a range of conditions near nominal were examined.  c. Test conditions representing the expected extreme conditions were examined.  d. Test conditions representing the entire range of conditions were examined.  **Measurements of Test Conditions**  a. Test conditions were qualitatively measured and/or characterized.  **b. One or more key characteristics of the test conditions were measured.**  c. All key characteristics of the test conditions were measured.  **Uncertainty of Test Condition Measurements**  a. Test conditions were not characterized or were characterized with gross observations; measurement uncertainty was not addressed.  b. Uncertainty analysis incorporated instrument accuracy only.  **c. Uncertainty analysis incorporated instrument accuracy and repeatability (i.e., statistical treatment of repeated measurements).**  d. Uncertainty analysis incorporated a comprehensive uncertainty quantification, which included instrument accuracy, repeatability, and other conditions affecting the measurements. |
| Assessment | **Equivalency of Input Parameters**  a. The types of some inputs were dissimilar.  b. The types of all inputs were similar, but the ranges were not equivalent.  **c. The types and ranges of all inputs were equivalent.**  **Output Comparison: Quantity**  a. A single output was compared.  **b. Multiple outputs were compared.**  **Output Comparison: Equivalency of Output Parameters**  a. Types of outputs were dissimilar.  b. Types of outputs were similar.  **c. Types of outputs were equivalent.**  **Output Comparison: Rigor of Output Comparison**  a. Visual comparison was performed.  b. Comparison was performed by determining the arithmetic difference between computational results and experimental results.  c. Uncertainty in the output of the computational model or the comparator was used in the output comparison.  **d. Uncertainties in the output of the computational model and the comparator were used in the output comparison.**  **Output Comparison: Agreement of Output Comparison**  a. The level of agreement of the output comparison was not satisfactory for key comparisons.  **b. The level of agreement of the output comparison was satisfactory for key comparisons, but not all comparisons.**  c. The level of agreement of the output comparison was satisfactory for all comparisons. |

**Table SA4.** Goals for credibility factors associated with applicability to the COU

| Applicability to COU | Relevance of the QOIs  a. The QOIs from the validation activities were related, though not identical, to those for the COU.  b. A subset of the QOIs from the validation activities were identical to those for the COU.  c. The QOIs from the validation activities were identical to those for the COU.  Relevance of the Validation Activities to the COU  a. There was no overlap between the ranges of the validation points and the COU.  b. There was partial overlap between the ranges of the validation points and the COU.  c. The COU encompassed some of the validation points.  d. The COU encompassed all validation and the validation points spanned the entire COU space. |
| --- | --- |

# APPENDIX B

Appendix B details the specific methodologies and comprehensive results of the Verification activities summarized in Sections 2.4 and 3.1 of the main text. This section comprises Code Verification, which confirms the credibility of the software utilized, and Calculation Verification, which evaluates the numerical accuracy of the developed model.

B1. Code Verification

Code verification is the stage that confirms whether the numerical implementation of the software is correct and if the developed code operates accurately as intended. This verification is performed through Software Quality Assurance (SQA) of the simulation software and Numerical Code Verification (NCV).

B1.1 Software Quality Assurance (SQA)

SQA is the process of verifying that the numerical implementation of the software functions correctly and produces reproducible results. In this study, the commercial software ANSYS 2024 R2 (ANSYS Inc., Canonsburg, PA, United States) was utilized. This software has maintained ISO 9001 certification, a quality management system standard, since 1995. This certification confirms that Ansys adheres to standardized procedures in the development, quality assurance, and verification services of its software products. Consequently, the pre-specified and documented SQA procedures of the simulation software serve as a critical basis for meeting the model credibility goals.

B1.2 Numerical Code Verification (NCV)

NCV was performed to ensure that the numerical implementation of the analysis software accurately converges to the mathematical analytical solution. This verification adhered to the recommendations of the ASME V&V 40 standard and simulated the four-point bending test defined in ASTM F382 (Figure SB1). The methodology involved a quantitative comparison between theoretical calculation results of four-point bending and FEA results obtained under identical geometric, material, and boundary conditions with loads ranging from 400 N to 1600 N. As presented in Table SB1, the error rate between the FEA simulation and the theoretical calculation results was found to be extremely low. The error rate for maximum deformation was consistently -0.021% across all load conditions, while the error rate for maximum normal stress was merely 0.704%. In conclusion, the maintenance of a maximum error rate of 0.704% demonstrates that the numerical implementation of the software used in this study sufficiently converges to the theoretical results.

**Figure SB1.** Geometric configuration for Numerical Code Verification. The benchmark model setup and boundary conditions used to verify the numerical implementation against analytical solutions.

Table SB1. Comparison of analytical solutions and Finite Element Analysis results for Numerical Code Verification

| Load (N) | Maximum deformation^a^ (mm) | | | Maximum normal stress^b^ (MPa) | | |
| --- | --- | --- | --- | --- | --- | --- |
|  | Analytical result | Simulation result | Error^*^(%) | Analytical result | Simulation result | Error^*^(%) |
| 400 | 1.062 | 1.062 | -0.021 | 976.563 | 983.436 | 0.704 |
| 800 | 2.124 | 2.123 | -0.021 | 1953.125 | 1966.871 | 0.704 |
| 1200 | 3.185 | 3.185 | -0.021 | 2929.688 | 2950.307 | 0.704 |
| 1600 | 4.247 | 4.246 | -0.021 | 3906.25 | 3933.743 | 0.704 |

* Error (%) = (Simulation result - Analytical result) / Analytical result × 100

a $Maximum deformation(mm): v_{\mathfrak{max}}=\frac{\left( P/2 \right)a}{24EI}\left( 3S^{2}-4a^{2} \right)$

b $Maximum normal stress(MPa): \sigma_{\max}=\frac{3Pa}{wh^{2}}$

B2. Calculation Verification

B2.1 Numerical Solver Error

Numerical solver error refers to result deviations caused by the selection of numerical parameters, such as the solver's Convergence Tolerance (CNVTOL). In this study, a problem-specific sensitivity study was conducted to verify whether its impact was negligible relative to the model's numerical accuracy goal (e.g., $\leq5\%$). The analysis environment utilized ANSYS Mechanical Static Structural. We quantitatively evaluated the impact by varying only the tolerance on the Flat 8H model under four-point static bending boundary conditions corresponding to ASTM F382 and a 5 mm displacement control condition. All settings other than the tolerance, including mesh, contact, materials, boundary conditions, loads, and substeps, were kept constant.

The convergence tolerance was set by switching Force/Moment/Displacement Convergence to user-defined mode in Nonlinear Controls. As shown in the figure below, the sensitivity analysis was performed by changing the convergence tolerance to 5%, which is ten times more relaxed than the reference value of 0.5% (Figure SB2). The relative change in reaction force between before (150.58 N) and after (151.74 N) the tolerance adjustment was calculated to be approximately 0.77%. Since this value is sufficiently small compared to the model's accuracy goal ($\leq5\%$), the impact of numerical solver tolerance changes on the QOIs was determined to be negligible. Therefore, the reference tolerance (Program Controlled) was maintained for all simulations in this analysis.

**Figure SB2.** Calculation Verification for Numerical Solver Error. Comparison of reaction forces between (left) program-controlled tolerance settings and (right) adjusted tolerance settings (relaxed by a factor of 10) to confirm negligible solver error.

B2.2 Use Error

To evaluate model input errors, an internal peer review involving five colleagues was conducted to verify all key inputs (geometry, boundary conditions, material properties, etc.) and outputs. This activity satisfies the criterion "c. Key inputs and outputs were verified by internal peer review" among the credibility factor grades defined in Appendix A. This verification process confirmed the absence of errors in the model setup.

# APPENDIX C

Appendix C details the methodology and results of the sample size determination for the uncertainty quantification summarized in Sections 2.5.3 and 3.2.3 of the main text.

**C1. Sample Size for Latin Hypercube Sampling**

In Latin Hypercube Sampling the range of each input parameter is divided into a number of equiprobable strata equal to the number of samples, and one value is drawn from each stratum. A design of n points therefore provides n strata per parameter. To determine an appropriate sample size for the three uncertain input parameters (E, a, h), designs of n = 10, 20, and 30 were generated by the same procedure and evaluated in sequence. For each design, FEA was performed at every design point under the boundary conditions used in the main analysis. The assessment was carried out for the bending stiffness, the yield force, and the force at 10 mm displacement. The bending structural stiffness and the bending strength were not examined separately, as they are metrics derived directly from the bending stiffness and the yield force, and therefore exhibit identical relative behavior with respect to sample size.

The mean predictions of the three QOIs agreed across the three designs, with the 95% confidence intervals of the means sharing a common interval in each case (Figure SC1). Since the mean prediction is the quantity that enters the comparison error (E = S - D), this indicates that E is not appreciably affected by the number of design points. The change in the standard deviation, which is reported as $u_{input}$, diminished as the sample size increased (Table SC1). For the bending stiffness the standard deviation changed by -9.8% from n = 10 to n = 20 but by only +1.9% from n = 20 to n = 30, and for the force at 10 mm displacement the corresponding changes were -4.0% and -0.8%. For the yield force the change was of similar magnitude over both intervals (-5.6% and -5.1%), amounting in absolute terms to 0.08 N on a mean of 114 N. The effect on the validation metric was correspondingly small. The ratio $|E|/U_{val}$ was 0.628, 0.690, and 0.687 for the bending stiffness at n = 10, 20, and 30; 0.860, 0.859, and 0.862 for the yield force; and 2.205, 2.231, and 2.244 for the force at 10 mm displacement. The validation outcome for each QOI was therefore the same for all three designs. On this basis, n = 30 was selected for the uncertainty quantification reported in the main text.

**Figure SC1.** Mean predictions of the bending stiffness, yield force, and force at 10 mm displacement obtained from Latin Hypercube Sampling designs of n = 10, 20, and 30. Error bars denote the 95% confidence intervals of the mean; shaded regions indicate the interval common to all three designs.

**Table SC1.** Mean, standard deviation, and validation metric of the Quantities of Interest for Latin Hypercube Sampling designs of n = 10, 20, and 30

| QOIs | n | Mean (S) | SD ($\boldsymbol{u}_{\boldsymbol{input}}\boldsymbol{)}$ | $\mathbf{\vert}\boldsymbol{E}\mathbf{\vert/}\boldsymbol{U}_{\boldsymbol{val}}$ |
| --- | --- | --- | --- | --- |
| Bending stiffness | 10 | 58.891 | 1.129 | 0.628 |
|  | 20 | 58.885 | 1.018 | 0.690 |
|  | 30 | 58.904 | 1.038 | 0.687 |
| Yield force (P) | 10 | 114.316 | 1.607 | 0.860 |
|  | 20 | 114.217 | 1.517 | 0.859 |
|  | 30 | 114.153 | 1.440 | 0.862 |
| Force at 10 mm | 10 | 136.525 | 0.922 | 2.205 |
|  | 20 | 136.519 | 0.885 | 2.231 |
|  | 30 | 136.492 | 0.878 | 2.244 |
